# Describing a “mass shooting”: the role of databases in understanding burden

**DOI:** 10.1186/s40621-019-0226-7

**Published:** 2019-12-03

**Authors:** Marisa Booty, Jayne O’Dwyer, Daniel Webster, Alex McCourt, Cassandra Crifasi

**Affiliations:** Department of Health Policy and Management, Center for Gun Policy and Research, Johns Hopkins Bloomberg School of Public Health, 624 N. Broadway, Baltimore, MD 21205 USA

**Keywords:** Mass shootings, Firearms, Gun violence

## Abstract

**Background:**

The mass shooting phenomenon has gained much attention lately as this form of gun violence appears to increase in frequency. Although many organizations collect information on mass shootings (fatal and nonfatal injuries), no federal definition of this phrase exists. The purpose of this study was to highlight the different statistics that result among databases that define and track “mass shootings.” Establishing definitive guidelines for a mass shooting definition could improve research credibility when presenting evidence to policy makers.

**Methods:**

We obtained data for mass shootings that occurred in 2017 from four sources: Gun Violence Archive, Mother Jones Investigation, Everytown for Gun Safety, and FBI’s Supplemental Homicide Report. We also examined FBI’s Active Shooter Report to compare the mass shootings datasets with active shooter situations, which have been federally defined. First, we examined the overlap among databases. Then, we applied the strictest fatal mass shooting definition to the mass shooting datasets to determine whether the differences in databases could be contributing to differences in fatalities and injuries recorded.

**Results:**

Gun Violence Archive recorded the most mass shooting incidents at 346 incidents in 2017, while Mother Jones only recorded 11 cases. Only 2 events were found in all four mass shooting datasets. When the strictest definition – four or more individuals fatally shot – was applied to all datasets, the number of mass shootings in 2017 ranged from 24 (Gun Violence Archive) to 5 (Mother Jones), but incidents collected still varied.

**Conclusions:**

There is much variety in statistics obtained from the different sources that have collected mass shooting information, with little overlap among databases. Researchers should advocate for a standard definition that considers both fatalities and nonfatalities to most appropriately convey the burden of mass shootings on gun violence.

## Background

In the aftermath of high profile, public mass shootings, news media outlets are saturated with coverage on both the specific event and the greater phenomenon of mass shootings in the United States. A study looking at mass shooting coverage in the New York Times found that among 90 mass shootings that occurred between 2000 and 2012, an average of 6 articles were written per mass shooting event (Schildkraut et al. 2018). Often included in media coverage are statistics regarding the frequency of mass shootings, average numbers of individuals killed per mass shooting for that year, and numbers of total mass shooting victims for the year thus far. However, these quickly-dispersed and widely cited statistics are heavily influenced – and subject to change – by the database from which they were generated.

Currently, no legal definition of the term “mass shooting” exists (Bagalman et al. 2013; Nichols 2017). The Federal Bureau of Investigation provides a close definition with the term “mass murder,” which is defined as “a number of murders (three or more) occurring during the same incident, with no distinctive time period between murder” (Federal Bureau of Investigation 2008). In response to the Sandy Hook Elementary school shooting, President Obama mandated a definition for “mass killing” with the Investigative Assistance for Violent Crimes Act of 2012, which defined this phrase as “3 or more killings” during an incident, excluding the death of the perpetrator (Investigative Assistance for Violent Crimes Act of 2012 2013). The Investigative Assistance for Violent Crimes Act also gave the FBI the authority to assist with investigations in which a mass killing took place. Mass shootings, however, are a specific subtype of “mass murders” or “mass killings” that denote use of a firearm during the incident. Neither a profile for mass shooting perpetrators nor a definition for mass shootings has been established, and different definitions of what constitutes a “mass shooting” lead to contradictory results between studies. For example, Adam Lankford completed a quantitative analysis of mass shootings that occurred between 1966 and 2012 and found that 31% of all mass shootings (four or more people killed) were perpetrated in the US (Lankford 2016). However, a more recent study by John Lott claimed that Lankford severely underestimated the mass shooting estimates for countries outside the US. Using data from the University of Maryland’s Global Terrorism Database, Lott found the US accounted for less than 1.43% of public mass shootings, a drastic decrease from the 31% estimated by Lankford (Lankford 2016; Lott 2018). Further investigation of Lott’s results by the Washington Post found that some of the discrepancies between Lankford’s and Lott’s findings were due to Lott’s inclusion of terrorism-related cases (Kessler 2018). Once these cases were removed from the analysis, Lott’s results more closely resembled Lankford’s (Kessler 2018). This is just one example that shows how using different mass shooting definitions – one that included terrorism incidents and one that did not – can lead to widely varied results and challenges in data interpretation.

Mass shootings continue to occur, and still no official definition of this phenomenon exists upon which researchers may rely. In the absence of a firm definition or systematic data collection by a federal agency, mass shooting databases have been created as an attempt to collect data and study these events. These databases track frequency, victim counts, and perpetrator characteristics, though the definitions of what constitutes a mass shooting are database-specific. This leads to discrepancies in mass shooting statistics, as exampled above by the contrast in the results of the Lankford and Lott papers. Discrepancies can lead to distrust of information, which hinders actions to reduce mass shootings. Policy makers may be compelled to enact legislation by advocates who claim that a mass shooting occurs almost every day of the year. However, that argument - and the gun violence legislation cause - would be significantly weakened if opponents presented data that portrayed mass shootings as a rare occurrence. Instead of passing legislation that could save countless lives and prevent numerous injuries, policymakers would be tied up sorting through the contradicting information to determine which statistics are most truthful. Enacting legislation is made more difficult when, as far as mass shootings are concerned, we have not defined what constitutes the “truth.”

To identify and characterize discrepancies that occur when different definitions are used for mass shootings, this paper examined five databases that collect mass shooting information. This research contributes to the literature by examining the effect that different definitions of “mass shooting” have on the reported statistics of mass shootings. Previous literature has compared different media sources’ reporting on mass shootings that meet specific victim criteria (Huff-Corzine et al. 2013), but there is a gap in research literature regarding differences in mass shooting definitions and how that changes reported statistics. Because mass shooting statistics are often used to promote and inform policy change, understanding the current working definition and how it influences reported statistics is critical to the conversation around mass shootings. Rather than multiple sources of conflicting data, there should be one working database with a widely agreed-upon definition. The goal of this analysis of different databases is to shed light on the nuances of how a “mass shooting” is currently defined by multiple entities and how that can influence estimates of the burden of mass shootings in the U.S.

## Methods

We examined what constitutes a “mass shooting” according to each of four data sources: Gun Violence Archive (GVA), Everytown for Gun Safety (EGS), Mother Jones Investigation, and the Federal Bureau of Investigation’s (FBI's) Supplementary Homicide Report (SHR). We also included the FBI's record of active shooter incidents. “Active shooter” and “mass shooting” are not synonymous, but the FBI’s record of active shooter incidents is the closest to federal data collection on events that resemble mass shooting incidents. First, we completed descriptive analyses of mass shootings that occurred in 2017 to illustrate the difference in statistics that may be reported depending on which dataset is referenced. We then evaluated database overlap by examining the events that were common to all or multiple databases. Finally, we applied one mass shooting definition- based on 4-person fatality count- to each of the 4 mass shooting data sources – excluding the active shooter report - to determine whether discrepancies among databases is accounted for when the same victim count-related definition is applied.

### Data sources

Data were obtained from four sources: Everytown for Gun Safety (2017), Gun Violence Archive (2015), the FBI’s Supplementary Homicide Report (Federal Bureau of Investigation 2018), and “U.S. Mass Shootings, 1982 – 2018” from Mother Jones’ Investigation (Mark Follman and Pan 2018). Information from the FBI’s Active Shooter Report for 2016–2017 (Schweit 2016) was also included in our study. Specifically, data on shootings that occurred in 2017 were collected, since this is the most recent year for which data are available from all sources. Definitions of “mass shooting” and collection requirements for each of the five databases are described below.

#### Everytown for gun safety

In 2018, Everytown for Gun Safety released a report on mass shootings that occurred from 2009 to 2017. According to Everytown, a mass shooting is an event in which at least four individuals are killed with a firearm; the fatality requirement excludes the shooter (Everytown for Gun Safety 2017). This definition is based on the 2005 FBI report on serial murder, and information included in Everytown’s analysis is pulled from media reports, official records, and the FBI’s Supplementary Homicide Report. When available, the EGS report included information on category of firearm(s) used and how the firearm(s) were acquired, whether the perpetrator was legally prohibited from owning firearms or could have been subjected to a gun violence restraining order (GVRO) law, and whether the location of the incident was a designated “gun-free” zone. Everytown collected information on incidents that occurred in both private and public settings, regardless of motive. Therefore, domestic violence incidents that occurred in the home may be included, as well as incidents of group violence pertaining to gang affiliation or drug trafficking.

#### Gun violence archive

Gun Violence Archive (GVA) is a non-profit organization established in 2013. The goal of GVA is to collect information regarding gun violence and to make this information available to the public online for use in gun violence and safety research. GVA defines a mass shooting as an event where four or more individuals are shot, but not necessarily killed (Gun Violence Archive 2015). Although this threshold is not supposed to include the shooter, exploratory analysis found that some of the shootings recorded did include the suspect in the count of people injured or killed. GVA differs from other datasets in that gang- and drug-related incidents were included in the GVA dataset. Data regarding mass shooting events were gathered via media and police sources, and links to media articles are provided for most mass shootings listed on the website. Information regarding shooter demographics, type of firearm, and venue of shooting may be listed on an individual shooting’s page or in links related to the shooting, but GVA does not necessarily track this information.

#### FBI’s supplementary homicide report

The FBI’s Uniform Crime Reporting (UCR) program was created in 1929 to be a collection of national crime statistics, and many researchers use the UCR to analyze crime. Reporting crime incidents to the UCR is not mandatory, and so UCR data rely on crime reporting from individual police agencies. Not all states choose to report to the UCR, and not even all agencies within a state choose to report crime information. The Supplementary Homicide Report (SHR) is a subset of the UCR that provides extensive details on homicides that have been reported, such as perpetrator demographics, weapon information, and incident circumstances. Although the FBI does not specifically label incidents as “mass shootings,” a few studies have been completed which use FBI SHR data to examine mass shooting trends (Reeping et al. 2019; Fox and Fridel 2016). For this reason, we have included an examination of the FBI SHR in this paper, with a definition of 4 or more killed in an incident as used in other publications.

#### Mother Jones’ investigation: US mass shootings, 1982–2018

The Mother Jones “Mass Shootings in America” is an open-source database that was created after the 2012 movie theater shooting in Aurora, Colorado. When Mother Jones initially collected data on mass shootings in 2012, they made use of the FBI’s definition for mass murder - four or more individuals killed in a single attack in a public place (Mark Follman and Pan 2018). However, the FBI’s definition was adjusted in 2013 – via mandate by President Obama - to include three or more people killed indiscriminately, and Mother Jones adjusted its definition and included qualifying incidents accordingly. Mother Jones does not include gang-related crimes or robbery in its dataset, and the shootings included were carried out indiscriminately by one individual in a public place. Information on venue, shooter demographics, shooter’s history of mental illness and weapon details are recorded in the Mother Jones dataset as well. Mother Jones admits that their data are narrowly focused and not as useful as other sources for studying gun violence as a whole, but they claim that their research provides insight at the “distinct phenomenon” of mass shootings (Mark Follman and Pan 2018).

#### FBI active shooter incidents in the United States in 2016 and 2017

In 2014, the FBI released a report detailing active shooter incidents that occurred between 2000 and 2013. The report was generated as an attempt to prepare law enforcement agencies for such situations and provide information that may help prevent incidents from occurring; an updated report has been released every 2 years since the release of the initial report in 2013. The FBI report defines an active shooter incident as a case in which at least one person is “actively engaged in killing or attempting to kill people in a populated area” (Schweit 2016). There is no set number of victims needed for an incident to be included in the FBI’s active shooter analysis. The FBI Active Shooter Report does not include shooting incidents that pertained to drug or gang involvement and accidental firearm discharges in public spaces were omitted from their analysis.

### Analytic strategy

For each dataset, descriptive statistics were generated using Stata 14.2 software to illustrate the fatality and victim counts and averages from mass shootings that occurred in 2017 (StataCorp 2015). The goal of the analysis was to highlight the differing findings that result when we compare multiple data sources, which all claim to track mass shootings (or in the FBI’s case, active shootings). After determining descriptive statistics for each dataset, we then excluded incidents with less than 4 people fatally injured; this is the definition used by Everytown and is the strictest victim-count definition out of the data sources examined. The purpose of this exclusion was to investigate whether applying the same definition to all the datasets would cause them to have similar numbers of mass shooting frequency and discuss any differences that may persist after the one definition is applied to all sources. To further highlight differences in the incidents captured among the examined databases, we used the R programming software to generate Venn Diagrams (Larsson 2019; R Core Team 2019).

## Results

### Descriptive statistics

Table 1 summarizes the frequency of mass shootings and the number of fatalities and injuries for each database. In 2017, GVA recorded the highest number of mass shootings at 346 incidents, whereas Mother Jones recorded the lowest number at 11 incidents. The FBI SHR counted the fewest total fatalities at 109 individuals, and GVA counted the most fatalities at 437 individuals. GVA had the fewest average victims per shooting at 6.5 victims injured or killed. The FBI SHR does not collect information on injuries that may have occurred with a homicide, so we cannot determine total victims in mass shooting incidents with this dataset. It is worthwhile to note that the Las Vegas shooting, which recorded over 500 casualties, is responsible for the high standard deviations.

**Table 1:** Mass Shooting Summary Statistics by Database

2017	Minimum Victims for Inclusion	Number of Shootings	Total Fatalities	Total Injured	Total Number of Victims	Victims per Shooting
EGS	4 killed	18	160	452	612	36 .0 (±116.3)
FBI’s SHR	4 killed	22	109	–	–	–
GVA	4 injured	346	437	1803	2240	6.5 (±26.8)
Mother Jones	3 killed	11	117	587	704	55.1 (±148.1)
FBI’s Active Shooter Report	No minimum	28	137	580	715	24.0 (±93.7)

### Database overlap

**Fig. 1:**
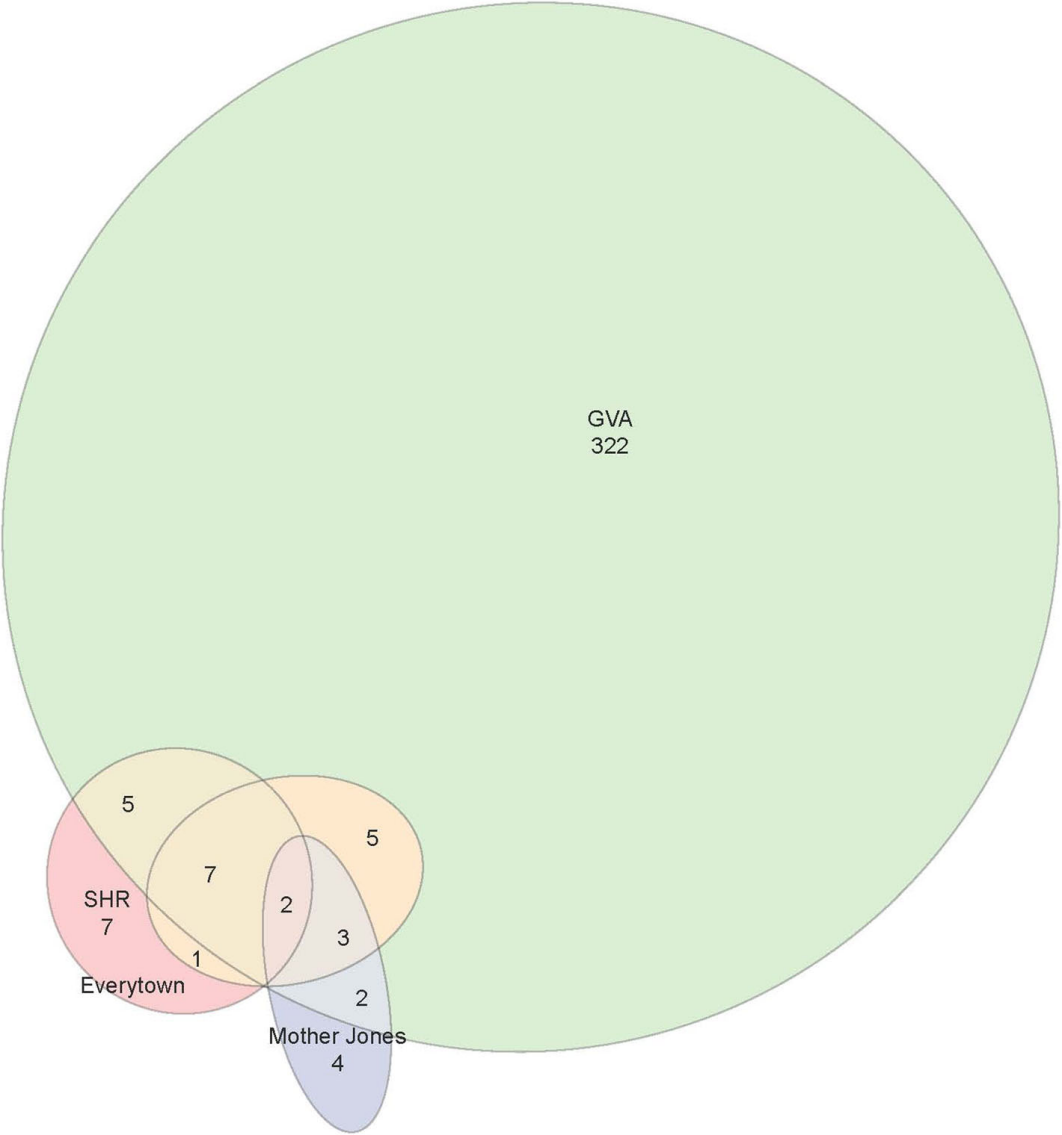
Venn Diagram of Database Overlap

Figure 1 is a Venn Diagram that illustrates the number of incidents that are common to the various databases. After examining the databases for potential overlap, only two shootings were found in all Gun Violence Archive, Everytown for Gun Safety, Mother Jones, and the FBI’s Supplemental Homicide Report. On October 1st, 58 individuals were fatally shot and over 440 individuals were injured when a man opened fire from the 32nd floor of Mandalay Bay Resort and Casino onto concert-goers below in Las Vegas, Nevada. On November 14th, 6 individuals were killed and 12 were injured by a during a shooting spree in Rancho Tehama, California. Both incidents involved high fatalities; the Las Vegas shooting is the deadliest shooting to occur in the history of the US. Also common to both events is the location. Each incident occurred in a public place: the crowded Las Vegas strip and around an elementary school. None of these events occurred in residential areas or could be attributed to drug or gang violence.

It is worthwhile to mention that three events were shared among the GVA, Mother Jones, and Everytown datasets, but were excluded from the SHR data. Two of the incidents occurred in Florida: one in a Fort Lauderdale airport where 5 individuals were killed and 6 were injured, and the other at a workplace in Orlando where 6 individuals were fatally shot. The third incident occurred in Sutherland Springs, Texas, when 27 people were fatally shot and 20 were injured when a gunman opened fire at a church. The SHR relies on police agencies’ reports of crime, and so we believe that the Florida incidents are absent because few Florida agencies report incidents to the FBI’s Uniform Crime Report. It is unclear why the Sutherland Springs shooting was absent from SHR data.

### Mass shooting frequencies with Everytown definition applied

**Fig. 2:**
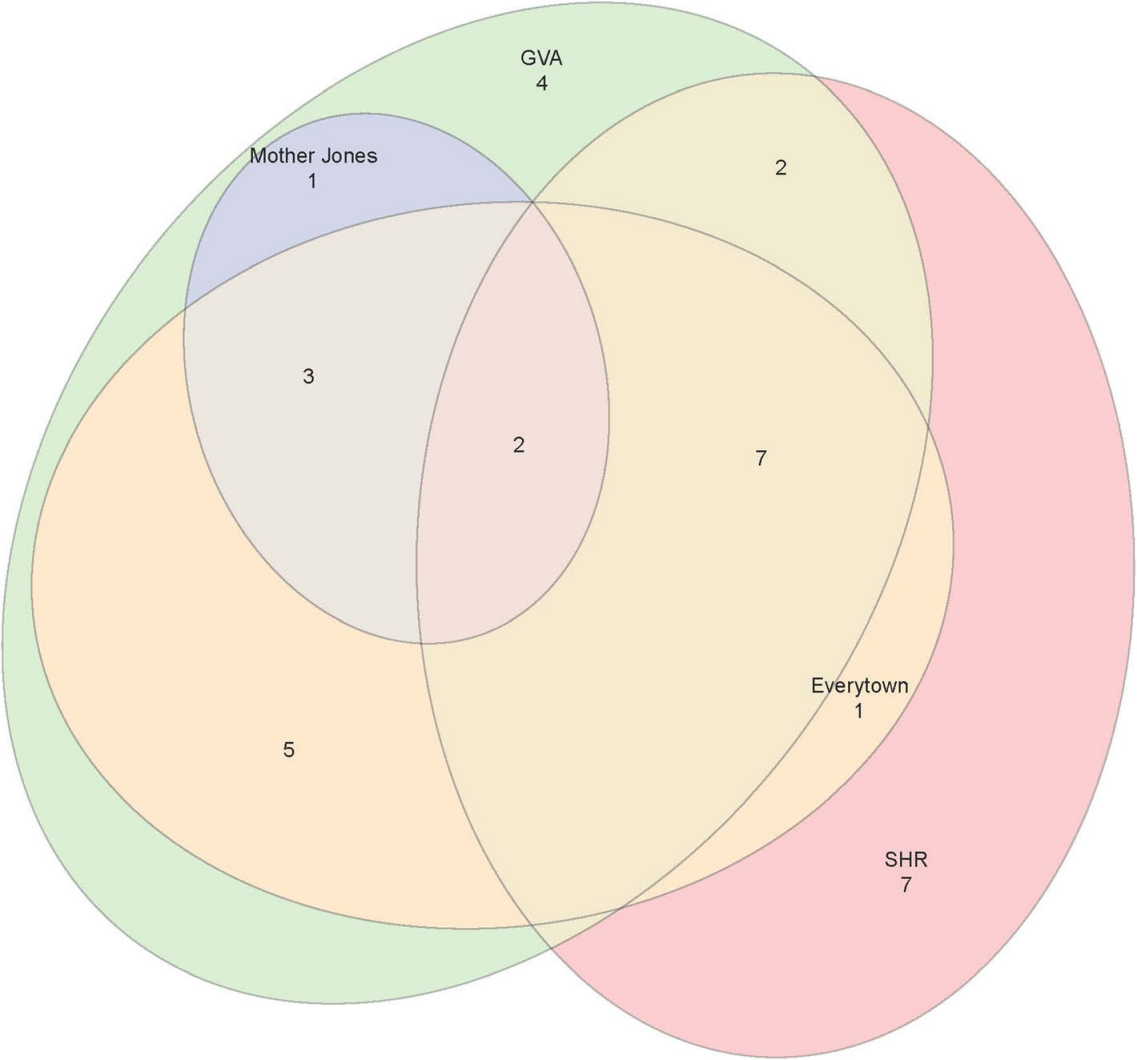
Venn Diagram of Database Overlap after “4+ Killed” is Applied to All

Table 2 provides the results of limiting all data sources to “four or more individuals killed.” After applying the “four or more individuals killed in an event” definition – the definition used by Everytown to track mass shootings – we have a minimum frequency of 5 shootings recorded by Mother Jones and a maximum of 24 shootings recorded by GVA. The Venn Diagram in Fig. 2 illustrates that even though the quantity of shootings has been made more similar, the content being captured is still very different. Although Everytown and the SHR both have the inclusion criterion of 4 or more fatalities, there are 4 more shootings included in the SHR than in Everytown’s analysis for 2017. Furthermore, only 10 incidents were common to both datasets; Everytown included 8 shootings not found in SHR’s data, and SHR included 12 shootings not found in Everytown’s analysis. This is similar for GVA in that the frequency of shootings is more similar, but the shooting incidents recorded are not the same. Upon closer examination, it appears that discrepancies in the information gathered about each shooting may lead to the some of the inconsistencies between databases. For example, the SHR records a shooting that occurred on May 14th, 2017 in Jonesboro, Arkansas in which 7 people were killed. However, this incident is recorded in GVA with 1 fatality and 6 injuries – we are confident that GVA is correct since they link to an article describing the shooting and cite 1 fatality, 6 injuries. (Staff 2017) Therefore, when eliminating incidents with less than 4 fatalities, this incident would be properly excluded from GVA and improperly included in the SHR counts of mass shootings.

**Table 2:** Frequency of Mass Shootings When “4+ Killed” (Everytown Standard) Is Applied

Database	Number of Mass Shootings (New Definition Applied)	Total Fatalities	Total Injuries	Number of Shootings Excluded from Original Dataset
Everytown	18	160	452	0 (same definition)
FBI’s SHR	22	109	–	0 (same definition)
GVA	24	191	486	322
Mother Jones	5	99	582	6

## Discussion

The results displayed a variety of answers to the descriptive questions that are commonly asked after a mass shooting takes place. A statistic as seemingly-straightforward as frequency of mass shootings in 2017 varied as widely as 11 to 346, depending on the definition used in the analysis.

Assessments of mass shootings currently hinge upon a researcher’s decision to follow the definition of a specific database. The definition determines what events are included, or rather what events are *not* included, thereby affecting any ensuing analysis. For example, using “public place” or “indiscriminate shooting” as criteria for defining mass shootings has the potential to exclude many cases where multiple people were shot at a place of residence. GVA notes instances of abusers following ex-partners to their homes and then killing the ex-partner, children, and current partner, but Mother Jones fails to capture such instances since these shootings are neither indiscriminate nor in a public place. Without a federal definition, researchers studying mass shootings need to be clear about what exactly they are examining. Are instances that may be spurred by domestic violence of interest? Does place of incident matter to your research question? These types of considerations must be specified explicitly when discussing mass shootings research.

There is great potential for media reporting bias in mass shootings. People who claim that a mass shooting occurs almost every day of the year are correct only by the standards of Gun Violence Archive. Individuals against the movement toward more comprehensive gun legislation would be more inclined to use the Mother Jones mass shooting data to endorse the rarity of such events, and therefore the lack of urgency needed in mass shooting prevention. Neither of the groups would have to manipulate data to fit their message – they simply need to choose the database with the definition that best fits their agenda. In this way, the absence of a standard mass shooting definition undermines high-quality research and reporting in a field that has been highly politicized.

With this in mind, we advocate for a definition of 4 or more casualties, without a restriction on location of incident or whether the incident had gang or drug involvement. Databases that define mass shootings by victim fatalities – rather than total number of victims injured or killed – fail to capture the injury caused when people survive gun violence. Individuals who are nonfatally shot in these incidents are discounted, though they may suffer physical and psychological traumas for the remainder of their lives. Restricting incidents to those that occurred in a public place undercounts the true number of events that result in mass shooting casualties, especially domestic violence incidents that occur in the home. We also urge researchers not to exclude incidents that appear to be gang- or drug-related because uninvolved bystanders are still being killed or injured in these events. If we fail to count gang- and drug-related incidents, then these incidents will be less likely to receive the same attention in terms of prevention efforts. For these reasons, we urge the federal government to establish a mass shooting definition of 4 or more casualties, excluding the perpetrator, regardless of place or gang- and/or drug-involvement.

The main limitation of this study was that data were sometimes unreliable, depending on the database being examined. For example, expired links to news articles on the GVA website impeded our ability to verify events when it seemed that the perpetrator was included in the victim count. Also, data from the SHR sometimes conflicted with data from other sources; an event found in SHR and another database would sometimes list information incorrectly in SHR. However, we believe that the major differences in mass shooting definitions among databases are still adequately highlighted. If anything, these issues with the datasets serve as more proof that we need a federal database that accurately captures information about these events.

## Conclusions

These findings highlight the need for a clear and consistent definition of a mass shooting that is cognizant of both fatalities and nonfatalities; information on the nature of the attack should be recorded, but not used as an exclusion criterion. Multiple sources should be used to corroborate events if data are collected from media sources, especially when considering the difficulty verifying some of the events listed as mass shootings in GVA. Without a clear and consistent definition, we lack the ability to build an adequate evidence base for potential interventions; results of effectiveness studies are going to greatly vary depending on which database is used. Establishing a definition for “mass shooting” will improve the quality of analyses being completed. This could lead to an improvement in not only public awareness and understanding of mass shooting facts, but also arguments to policymakers for legislation that could alleviate the burden that mass shootings place on society.

## Data Availability

The data analyzed in this study are available at Gun Violence Archive, https://www.gunviolencearchive.org/, Mother Jones’ Investigation, https://www.motherjones.com/politics/2012/12/mass-shootings-mother-jones-full-data/2018/, Everytown for Gun Safety, https://everytownresearch.org/reports/mass-shootings-analysis/, Federal Bureau of Investigations Uniform Crime Reporting Program, https://ucr.fbi.gov/crime-in-the-u.s/2017/crime-in-the-u.s.-2017/topic-pages/expanded-offense, and Federal Bureau of Investigations – Active Shooter Incidents in 2014 and 2015, https://www.fbi.gov/file-repository/activeshooterincidentsus_2014-2015.pdf/view.

## Abbreviations

EGS: Everytown for Gun Safety
FBI: Federal Bureau of Investigation
GVA: Gun Violence Archive
SHR: Supplementary Homicide Report
